# A Year in Review: Atrial Fibrillation 2023

**DOI:** 10.19102/icrm.2024.15017

**Published:** 2024-01-15

**Authors:** Amier Ahmad, Ankur A. Karnik, Rahul Doshi

**Affiliations:** 1 Department of Clinical Cardiac Electrophysiology, Scottsdale, AZ, USA

**Keywords:** Anticoagulation, atrial fibrillation, catheter ablation, guidelines, left atrial appendage occlusion

## Introduction

*“We have to reinvent the wheel every once in a while, not because we need a lot of wheels; but because we need a lot of inventors”—Bruce Joyce, author*

From updated clinical guidelines to groundbreaking science, 2023 delivered several new insights into the management of atrial fibrillation (AF). Here, we highlight the most important topics covered during the past year.

## Guideline updates

The American College of Cardiology (ACC) and the American Heart Association (AHA) have released updated guidelines for the prevention and management of AF.^1^ The ACC/AHA introduced a new staging classification, moving away from the prior emphasis on AF duration and instead focusing on AF being a progressive disease. The new staging criteria are as follows:

1. At risk for AF
2. Pre-AF (structural/imaging or electrical findings predisposing to AF)
3. AF (3A, paroxysmal; 3B, persistent; 3C, long-standing persistent; 3D, successful AF ablation)
4. Permanent AF

Furthermore, the guidelines emphasize early rhythm control, particularly in the management of reduced left ventricular ejection fraction (class 1 level of recommendation). The use of catheter ablation as a first-line level of therapy to reduce symptoms and the progression to persistent AF in select patients (younger age with fewer comorbidities) with paroxysmal AF was awarded a class 1 level of recommendation. The use of left atrial appendage occlusion devices (LAAODs) was also awarded a class 2a level of recommendation. Further updates in regard to the use of catheter ablation, anti-arrhythmic drugs (AADs), and anticoagulation were made based on recent publications and are highlighted below.

## Catheter ablation for atrial fibrillation

The hot-button topic this past year regarding catheter ablation has been the use of pulsed-field ablation (PFA). PFA is currently widely used in Europe and is being introduced in the United States. However, the results of the Randomized Controlled Trial for Pulsed Field Ablation versus Standard of Care Ablation for Paroxysmal Atrial Fibrillation (ADVENT) have provided data that may temper expectations. About 600 paroxysmal AF patients were randomized to either pulmonary vein isolation (PVI) using PFA or conventional therapy (radiofrequency or cryoablation).^2^ The primary composite endpoint included initial procedural failure, atrial tachyarrhythmia after a 3-month blanking period, the use of AADs, cardioversion, or repeat ablation. The primary safety endpoint included serious acute or chronic device/procedure-related adverse events. Similar results were seen in both groups at 1 year with respect to avoiding the primary endpoint (73.3% PFA vs. 71.3% conventional). The primary safety endpoint was also similar between the groups (2.1% PFA vs. 1.5% conventional). PFA was noted to lower the procedural time (105 vs. 123 min). There were no documented instances of atrio-esophageal fistula or pulmonary vein stenosis. Phrenic nerve injury occurred in both groups (4 PFA vs. 7 conventional). Serious adverse events were numerically similar (51 PFA vs. 52 conventional), with two patients in the PFA arm experiencing a pericardial effusion with tamponade (one fatal) versus no events of tamponade in the conventional group. PFA was deemed non-inferior, although the margin of non-inferiority was generous (15% in absolute terms). Overall, the safety profile of PFA was disappointing. Although touted as cardio-selective, instances of phrenic nerve injury occurred. Furthermore, a subset of patients underwent cerebral imaging to uncover the presence of silent infarcts, which were found in 3 of 33 patients in the PFA arm versus 0 of 37 patients in the conventional arm. Separately, results from a European registry published in the multicenter EUropean Real World Outcomes with Pulsed Field AblatiOn in Patients with Symptomatic AtRIAl Fibrillation (EU-PORIA) study showed slightly more promising results with a 1-year arrhythmia-free survival of 74% (80% paroxysmal and 66% persistent).^3^ However, 169 patients (14%) had additional lesions outside the pulmonary veins, most commonly in the posterior wall. The median procedural time was 58 min (interquartile range [IQR], 40–87 min). Major complications occurred in 1.7% of procedures (1.1% cardiac tamponade, 0.6% transient ischemic attack or stroke).

Non-randomized data based on the Pulsed Field Ablation to Irreversibly Electroporate Tissue and Treat AF (PULSED AF) trial showed similar efficacy of PFA in paroxysmal and persistent symptomatic AF patients.^4^ At 1 year, the primary effectiveness endpoint consisting of freedom from procedural failure, arrhythmia recurrence, or AAD escalation was found to be 66.2% in the paroxysmal AF group and 55.1% in the persistent AF group. The primary safety endpoint of freedom from serious procedure/device-related adverse events was low at 0.7%.

Time will tell whether the excitement behind PFA is justified. PFA appears to offer similar efficacy but may decrease the overall procedural time.

## Ablation techniques/populations of interest

The updated ACC/AHA guidelines emphasize the use of catheter ablation early in the treatment of AF.^1^ This raises the question of how early catheter ablation should be offered to patients. This topic was addressed by the Early Aggressive Invasive Intervention for Atrial Fibrillation (EARLY-AF) investigators in a randomized controlled trial assigning drug-naïve individuals with paroxysmal AF to either^5^ an initial rhythm control strategy with cryoballoon ablation or AADs.^5^ Over a period of 36 months of follow-up, 1.9% of patients in the ablation arm had an episode of persistent AF (an episode of AF lasting ≥7 days or lasting 48 h to 7 days but requiring cardioversion for termination) versus 7.4% in the AAD group. Recurrent atrial arrhythmias were higher in the AAD group (77.2% vs. 56.5%; 95% confidence interval [CI], 0.38–0.67). These findings suggest that early intervention may lower AF burden in the long run, but is there harm in delaying intervention? This question was addressed by researchers from Australia evaluating early versus delayed ablation with a primary endpoint of AF recurrence in the 12 months following ablation.^6^ Patients were randomized to AF ablation within 1 month of referral or delayed ablation after 12 months. No difference was seen in ablation efficacy, in addition to secondary outcomes such as AF burden or AAD use following ablation, after a 12-month delay. The trial included a low number of patients (48 early vs. 41 delayed) and a higher number of patients with persistent AF in the early arm (54% vs. 37%). As such, the results must be taken cautiously, but it is encouraging to note that efficacy outcomes may be stable for up to 1 year, allowing providers to maximize lifestyle changes that can influence AF dramatically.

The role of early AF intervention for patients with heart failure was also a hot topic this year. The Catheter Ablation for Atrial Fibrillation in patientS with End-sTage Heart Failure and Eligibility for Heart Transplantation (CASTLE HTx) investigators screened roughly 900 patients referred for a heart transplant or left ventricular assist device (LVAD) implantation who had symptomatic AF and randomized 194 patients to either AF ablation with PVI alone or medical therapy.^7^ The average left ventricular ejection fraction was 25% in the medical therapy group and 29% in the ablation group. The primary composite endpoint included all-cause death, LVAD implantation, or urgent heart transplantation. The study was terminated early after 1 year due to the substantial benefit seen in the ablation arm. After a median follow-up of 18 months, the primary endpoint was seen in 30% in the medical therapy arm versus 8% in the ablation arm (hazard ratio [HR], 0.29; 95% CI, 0.12–0.72). The findings of this study were met with skepticism. The early separation in the Kaplan–Meier curves differs from what was seen in the Catheter Ablation for Atrial Fibrillation with Heart Failure (CASTLE-AF) study, which did not see a mortality benefit for 3 years.^8^ Limitations to CASTLE HTx include a 16% crossover rate in the medical therapy arm and the existence of patients who may not have been in the “end stage” of disease (one-third of patients had New York Heart Association functional class II disease). Overall, clinicians should tailor treatment strategies for individual patients, as some may benefit from an early rhythm approach, while others may be better candidates for a watch-and-wait strategy.

High-power, short-duration (HPSD) radiofrequency ablation was another topic of interest in the past year. This technique has gained popularity due to reduced procedural times and theoretically improved safety during ablation. The safety and efficacy of this technique have been established in multiple observational studies. A Trial of High Power-Short Duration Versus Standard Power-Long Duration Radiofrequency Ablation for Treatment of Atrial Fibrillation (SHORT-AF) was a randomized trial evaluating a 50-W HPSD approach for catheter ablation of paroxysmal and persistent AF with PVI.^9^ Sixty patients were randomized to either an HPSD approach for every lesion or a standard approach of 25 W delivered to the posterior wall and 30–35 W anteriorly. The findings were consistent with those of previous observational studies showing a shorter median time from the first to the last lesion (87 min in the HPSD group vs. 126 min in the standard duration group, *P* = .003). The total procedural time was shorter in the HPSD group, albeit not statistically significantly (236 vs. 286 min). AF recurrence rates favored HPSD (10% vs. 35%). The only area of doubt was a greater rate of asymptomatic cerebral emboli (ACE) seen in the HPSD group (40% vs. 17%, *P* = .053). Although it did not show statistical significance, this finding is concerning. Multiple factors influence the rates of ACE, including ablation catheter type and intraprocedural activated clotting time, which makes assessing this finding difficult. Furthermore, the potential increase in ACE with HPSD needs to be balanced with the reduction in long-term AF burden, which is independently associated with ACE and potential dementia.^10^

A very HPSD (90 W/4 s) ablation technique was studied in the Very High Power Ablation in Patients with Atrial Fibrillation Schedule for a First Pulmonary Vein Isolation (POWER PLUS) multicenter, randomized controlled trial.^11^ A total of 180 patients with symptomatic or paroxysmal AF were randomized to undergo first-time PVI by either contiguous application of ablation lesions using a very HPSD technique or a standard 35/50-W application (titrated based on the location). There was a non-significant trend toward lower first-pass isolation in the HPSD group (83.9% vs. 90%, *P* = .0852). At 6 months of follow-up, similar recurrent arrhythmias were seen in both groups (17% HPSD vs. 15% standard, *P* = .681). Also, the procedural time was modestly reduced with HPSD (70 [IQR, 60–80] vs. 75 [IQR, 65–88.3] min, *P* = .009). No major complications were seen in either group. Despite the overall smaller number of randomized patients, the trial findings highlight the relative safety of this technique. The multicenter, non-randomized Q-FFICIENCY trial compared a very HPSD technique to a standard ablation technique (25–50 W) in patients with symptomatic paroxysmal AF.^12^ This trial utilized the upcoming Biosense Webster QDOT Micro catheter, which allows for an energy delivery up to 50 W for 60 s in a standard mode and 90 W for 4 s in a proprietary QMODE+. The trial found a rate of clinical success (defined as freedom from symptomatic recurrence) of 86.0% at 12 months. Further large-scale, randomized trials will be needed to truly determine the efficacy and safety of this technique.

## Anticoagulation

One of the most interesting questions from this past year was that of “what amount of AF warrants anticoagulation?” The Non–vitamin K antagonist Oral anticoagulants in patients with Atrial High rate episodes (NOAH-AFNET 6) trial evaluated the use of edoxaban (vs. placebo) in patients with short-duration atrial high-rate episodes (AHREs) detected on an implantable cardiac device (median duration, 2.8 h).^13^ This study was a double-blind, randomized trial with about 2500 patients (mean age, 78 years; mean CHA_2_DS_2_-VASc score, 4 points). The primary composite endpoint included stroke, systemic embolism, and cardiovascular death. Ultimately, the trial was terminated early after a median follow-up of 21 months due to a signal of harm. The primary endpoint occurred in 3.2% per patient-year in the edoxaban group (vs. 4.0% placebo; 95% CI, 0.60–1.08). The stroke rates were low in both groups (0.9% edoxaban vs. 1.0% placebo). Major bleeding or death occurred significantly more in the edoxaban group (5.9% vs. 4.5% per patient-year; 95% CI, 1.02–1.67). Subsequently, the Apixaban for the Reduction of Thrombo-embolism in Patients with Device-Detected Subclinical Atrial Fibrillation (ARTESiA) trial investigators randomized >4000 patients (mean age, 77 years; mean CHA_2_DS_2_-VASc score, 3.9 points) with short-duration (mean duration, 1.5 h), asymptomatic AF to apixaban or aspirin.^14^ The primary endpoint consisted of stroke and systemic embolism, which occurred in 0.78% per patient-year in the apixaban arm versus 1.24% in the aspirin arm (95% CI, 0.45–0.88). Major bleeding, as expected, was more common in the apixaban arm, with a relative risk increase of 80% (HR, 1.80; 95% CI, 1.26–2.57). A meta-analysis of these two studies supports the use of systemic anticoagulation to reduce the risk of stroke in subclinical AF, at the expense of major bleeding.^15^ The takeaway from these studies is that the risk of stroke conveyed by subclinical AF is low. The decision to anticoagulate remains nuanced, and a shared decision-making approach is ideal. The current ACC/AHA guidelines state the following:^1^

1. It is reasonable to consider systemic anticoagulation in patients with an AHRE lasting ≥24 h and with a CHA_2_DS_2_-VASc score of 2 points or equivalent stroke risk.
2. It is reasonable to consider systemic anticoagulation in patients with a device-detected AHRE lasting between 5 min and 24 h and with a CHA_2_DS_2_-VASc score of ≥3 points or equivalent stroke risk.
3. Anticoagulation should be deferred for those patients with an AHRE lasting <5 min without another indication for anticoagulation.

Similar to the uncertainty regarding anticoagulation strategies for AHREs, there remains a lack of clinical guidance regarding the initiation of anticoagulation for individuals with AF and an intermediate stroke risk (CHA_2_DS_2_-VASc score, 1 point). Investigators from the Atrial Fibrillation in Norway (AFNOR) study aimed to determine the clinical benefit of anticoagulation in this population.^16^ A total of 34,460 patients with a CHA_2_DS_2_-VASc score of 1 point were identified and followed up with until their first incidence of ischemic stroke, intracranial hemorrhage, an increase in CHA_2_DS_2_-VASc score, or the study period ended. Anticoagulation was associated with a lower combined endpoint of ischemic stroke, major bleeding, and mortality (adjusted HR, 0.57; 95% CI, 0.51–0.63). Although interesting, these data do not account for bleeding episodes that do not lead to hospitalization but remain a significant cause of concern for patients (ie, nosebleeds, bruising).

This year saw the rise of abelacimab, a factor XI inhibitor, as a promising new anticoagulant with the potential to prevent thrombosis, while preserving hemostasis. The Safety and Tolerability of Abelacimab (MAA868) vs. Rivaroxaban in Patients with Atrial Fibrillation (AZALEA-TIMI 71) (NCT04755283) study is a phase 2b study comparing bleeding rates between patients with AF given abelacimab or rivaroxaban. The trial was stopped early due to a significantly greater-than-expected bleeding reduction in the abelacimab arm. A higher rate of ischemic stroke was seen with abelacimab compared to rivaroxaban, although the trial was not powered to draw conclusions regarding strokes. Despite these promising results, early results from the Phase 3 Study to Compare the Efficacy and Safety of the Oral FXIa Inhibitor Asundexian with Apixaban for the Prevention of Stroke or Systemic Embolism in Male and Female Participants Aged 18 Years and Older with Atrial Fibrillation at Risk for Stroke (OCEANIC AF) trial (NCT05643573) investigating asundexian versus apixaban in patients with AF are sobering. The trial has been stopped early due to the inferior efficacy of the investigational drug for stroke prevention compared to the control arm. We will have to wait for the full publication of both trials to appraise the data, but there does seem to be a chink in the armor of factor XI inhibitors.

## Left atrial appendage occlusion devices

This past year provided additional information regarding the use of LAAODs. The current guidelines awarded LAAODs a higher-level class of recommendation: in patients with a CHA_2_DS_2_-VASc score of ≥2 points and a contraindication to long-term oral anticoagulation (OAC), LAAODs are considered reasonable (class 2a). In patients with a moderate-to-high risk of stroke and a high risk of major bleeding on OAC, LAAODs may be a reasonable alternative to OAC (class 2b).^1^ Despite a stronger recommendation, the 1-year results of the Comparison of Amulet Versus Watchman/FLX Device in Patients Undergoing Left Atrial Appendage Closure (SWISS-APERO) trial published this past year may facilitate a pause. The main trial was a multicenter, randomized controlled trial of 221 patients randomized to either the Amulet (Abbott, Chicago, IL, USA) or WATCHMAN FLX (Boston Scientific, Marlborough, MA, USA) devices.^17^ At 1 year of follow-up, the patency rate was 53.6% in patients with Amulet devices and 48.8% in patients with WATCHMAN devices.^18^ A potential cause for the high patency rate may have been the use of computed tomography to assess patency versus transesophageal echocardiography. The clinical consequences of a peridevice leak (PDL) remain controversial. Based on the 5-year study outcomes, a PDL of ≤5 mm, traditionally considered acceptable, conferred higher rates of thromboembolism in patients who had undergone WATCHMAN placement.^19^ These findings raise the question of how often an assessment for PDL is required following LAAOD implantation.

## Conclusion

The year 2023 did not disappoint, with several thought- provoking and clinically relevant studies published during its course. There remain a plethora of upcoming trials that should make 2024 even more interesting.

## References

[1] Joglar JA, Chung MK, Armbruster AL 2023 ACC/AHA/ACCP/HRS guideline for the diagnosis and management of atrial fibrillation: a report of the American College of Cardiology/American Heart Association Joint Committee on Clinical Practice Guidelines [published online ahead of print November 30, 2023]. Circulation.

[2] Reddy VY, Gerstenfeld EP, Natale A (2023). Pulsed field or conventional thermal ablation for paroxysmal atrial fibrillation. N Engl J Med.

[3] Schmidt B, Bordignon S, Neven K (2023). European real-world outcomes with Pulsed field trialn in patients with symptomatic trial fibrillation: lessons from the multi-centre EU-PORIA registry. Europace.

[4] Verma A, Haines DE, Boersma LV (2023). Pulsed field ablation for the treatment of atrial fibrillation: PULSED AF pivotal trial. Circulation.

[5] Andrade JG, Deyell MW, Macle L (2023). Progression of atrial fibrillation after cryoablation or drug therapy. N Engl J Med.

[6] Kalman JM, Al-Kaisey AM, Parameswaran R (2023). Impact of early vs. delayed atrial fibrillation catheter ablation on atrial arrhythmia recurrences. Eur Heart J.

[7] Sohns C, Fox H, Marrouche NF (2023). Catheter ablation in end-stage heart failure with atrial fibrillation. N Engl J Med.

[8] Marrouche NF, Brachmann J, Andresen D (2018). Catheter ablation for atrial fibrillation with heart failure. N Engl J Med.

[9] Lee AC, Voskoboinik A, Cheung CC (2023). A randomized trial of high vs standard power radiofrequency ablation for pulmonary vein isolation: SHORT-AF. Clin Electrophysiol.

[10] Bunch TJ (2020). Atrial fibrillation and dementia. Circulation.

[11] O’Neill L, El Haddad M, Berte B (2023). Very high-power ablation for contiguous pulmonary vein isolation: results from the randomized POWER PLUS trial. JACC Clin Electrophysiol.

[12] Osorio J, Hussein AA, Delaughter MC (2023). Very high-power short-duration, temperature-controlled radiofrequency ablation in paroxysmal atrial fibrillation: the prospective multicenter Q-FFICIENCY trial. JACC Clin Electrophysiol.

[13] Kirchhof P, Toennis T, Goette A (2023). Anticoagulation with edoxaban in patients with atrial high-rate episodes. N Engl J Med.

[14] Healey JS, Lopes RD, Granger CB Apixaban for stroke prevention in subclinical atrial fibrillation. [published online ahead of print November 12, 2023]. N Engl J Med.

[15] Mcintyre WF, Benz AP, Becher N Direct oral anticoagulants for stroke prevention in patients with device-detected atrial fibrillation: a study-level meta-analysis of the NOAH-AFNET 6 and ARTESiA trials [published online ahead of print November 12, 2023]. Circulation.

[16] Anjum M, Ariansen I, Hjellvik V Stroke and bleeding risk in atrial fibrillation with CHA2DS2-VASC risk score of one: the Norwegian AFNOR study [published online ahead of print November 23, 2023]. Eur Heart J.

[17] Galea R, De Marco F, Meneveau N (2022). Amulet or watchman device for percutaneous left atrial appendage closure: primary results of the SWISS-APERO randomized clinical trial. Circulation.

[18] Galea R, Meneveau N, De Marco F One-year outcomes after amulet or watchman device for percutaneous left atrial appendage closure: a pre-specified analysis of the SWISS-APERO randomized clinical trial [published online ahead of print October 24, 2023]. Circulation.

[19] Reddy VY, Doshi SK, Kar S (2017). 5-Year outcomes after left atrial appendage closure: from the PREVAIL and PROTECT AF trials. J Am Coll Cardiol.

